# Supplementary material for: Joint Developmental Trajectories of Perinatal Depression and Anxiety and Their Predictors: A Longitudinal Study
Source: Healthcare (Basel). 2025 May 26;13(11):1251. doi: 10.3390/healthcare13111251 (PMC12154182; doi:10.3390/healthcare13111251)
Supplement: Supplementary file 1 [file healthcare-13-01251-s001.zip › healthcare-3527434-supplementary.pdf]

Supplementary:

Table S1. Means, standard deviations and correlation analysis of variables (n=1062)

	M ± SD	Dep T1	Dep T2	Dep T3	Dep T4	Anx T1	Anx T2	Anx T3	Anx T4	Social support	Family support	Friend support	Other support
Dep T1	3.37±3.79	1											
Dep T2	3.08±3.71	0.894**	1										
Dep T3	3.59±3.85	0.854**	0.876**	1									
Dep T4	4.07±4.22	0.802**	0.829**	0.866**	1								
Anx T1	3.15±3.25	0.697**	0.688**	0.689**	0.644**	1							
Anx T2	2.72±2.91	0.686**	0.694**	0.696**	0.640**	0.829**	1						
Anx T3	3.10±3.21	0.747**	0.751**	0.790**	0.710**	0.812**	0.829**	1					
Anx T4	2.81±3.12	0.664**	0.672**	0.706**	0.693**	0.762**	0.766**	0.832**	1				
Social support	70.08±8.62	-0.737**	-0.739**	-0.750**	-0.778**	-0.588**	-0.591**	-0.661**	-0.602**	1			
Family support	24.03±3.75	-0.677**	-0.674**	-0.688**	-0.704**	-0.541**	-0.545**	-0.601**	-0.549**	0.922**	1		
Friend Support	23.54±3.09	-0.711**	-0.730**	-0.739**	-0.760**	-0.578**	-0.588**	-0.659**	-0.609**	0.956**	0.863**	1	
Other support	22.51±2.72	-0.595**	-0.584**	-0.590**	-0.634**	-0.461**	-0.455**	-0.517**	-0.459**	0.813**	0.563**	0.705**	1

Note: ** $p < 0.01$, Dep represents Depression, Anx represents Anxiety.

Table S2. Fit indices of latent variable growth models for perinatal depression and anxiety

	Model	χ^2(df)	P	CFI	TLI	RMSEA (90% CI)	SRMR	BIC	Coefficients			Variances		
									Intercept	Slope	Slope of curves	Intercept	Slope	Slope of curves
Dep	Linear	134.294(5)	<0.001	0.974	0.969	0.156 (0.134,0.179)	0.029	18827.994	3.177***	0.225***		12.663***	0.287***	
	Quadratic	23.283(1)	<0.001	0.995	0.973	0.145 (0.098,0.199)	0.013	18744.855	3.340***	-0.355***	0.205***	13.155***	0.515	0.062
	Free estimated	22.210(3)	<0.001	0.996	0.992	0.078 (0.049,0.109)	0.017	18729.846	3.262***	-0.15**		12.671***	0.065	
Anx	Linear	135.568(5)	<0.001	0.967	0.961	0.157 (0.135,0.180)	0.046	17914.589	3.041***	-0.065**		8.421***	0.177***	
	Quadratic	77.674(1)	<0.001	0.981	0.885	0.269 (0.220,0.321)	0.042	17884.567	2.916***	-0.164*	0.031	5.539***	-3.104***	0.046
	Free estimated	33.757(3)	<0.001	0.992	0.985	0.098 (0.070,0.129)	0.016	17826.714	3.136***	-0.433***		8.545***	-0.607**	

Note: * $p < 0.05$, ** $p < 0.01$, *** $p < 0.001$.

Table S3. Parameter information of latent class growth models for perinatal depression and anxiety

	Class	n (%)	Intercept estimated mean	Slope estimated mean
Dep	C1 Moderate risk depression group	186, 17.5%	7.271***	-0.509***
	C2 Low risk depression group	204, 19.2%	3.891***	-0.312**
	C3 Consistently low depression group	596, 56.1%	0.732***	-0.147**
	C4 High risk depression group	76, 7.2%	12.117***	0.081
Anx	C1 Consistently low anxiety group	539, 50.8%	0.823***	-0.145*
	C2 Low risk anxiety group	318, 29.9%	3.745***	-0.58***
	C3 High risk anxiety group	50, 4.7%	11.191***	-1.297***
	C4 Moderate risk anxiety group	155, 14.6%	7.072***	-0.638**

Note: * $p < 0.05$, ** $p < 0.01$, *** $p < 0.001$.

Table S4. Fit indices of parallel process latent growth models for perinatal depression and anxiety

Model	χ^2(df)	P	CFI	TLI	RMSEA (90% CI)	SRMR	BIC	Coefficients						Variances					
								Intercept (dep)	Slope(dep)	Slope of curves (dep)	Intercept (anx)	Slope (anx)	Slope of curves(anx)	Intercept (dep)	Slope (dep)	Slope of curves	Intercept (anx)	Slope (anx)	Slope of curves

																(dep)			(anx)
Linear	341.219(22)	<0.0001	0.969	0.960	0.117 (0.106,0.128)	0.033	35589.905	3.159***	0.168***		2.987***	-0.019		12.550***	0.134***		8.073***	0.072***	
Quadratic	197.99(9)	<0.0001	0.981	0.942	0.141 (0.124,0.158)	0.029	35537.268	3.357***	-0.341***	0.134***	3.018***	-0.099	0.019	13.558***	0.585*	0.001	8.061***	-0.049	-0.001
Free estimated	190.604(18)	<0.0001	0.983	0.974	0.095 (0.083,0.107)	0.02	35467.161	3.257***	-0.144**		3.132***	-0.428***		12.609***	0.054		8.574***	-0.586*	

Note: * $p < 0.05$, ** $p < 0.01$, *** $p < 0.001$, Dep represents Depression, Anx represents Anxiety.

Table S5. Mean and standard deviation of perinatal depression and anxiety in different developmental classes (M±S)

Class	Dep (T1)	Dep (T2)	Dep (T3)	Dep (T4)	Anx (T1)	Anx (T2)	Anx (T3)	Anx (T4)
1 (n=33)	15.39±2.82	13.61±1.98	11.45±2.76	10.06±2.02	9.91±3.46	9.03±3.16	10.36±3.70	8.36±2.35
2 (n=754)	1.49±1.82	1.16±1.52	1.49±1.70	1.87±1.98	1.87±2.19	1.59±1.91	1.70±1.84	1.61±1.97
3 (n=275)	7.09±2.17	7.07±2.67	8.41±2.36	9.40±2.39	5.84±3.00	5.04±2.81	6.07±2.91	5.43±3.37

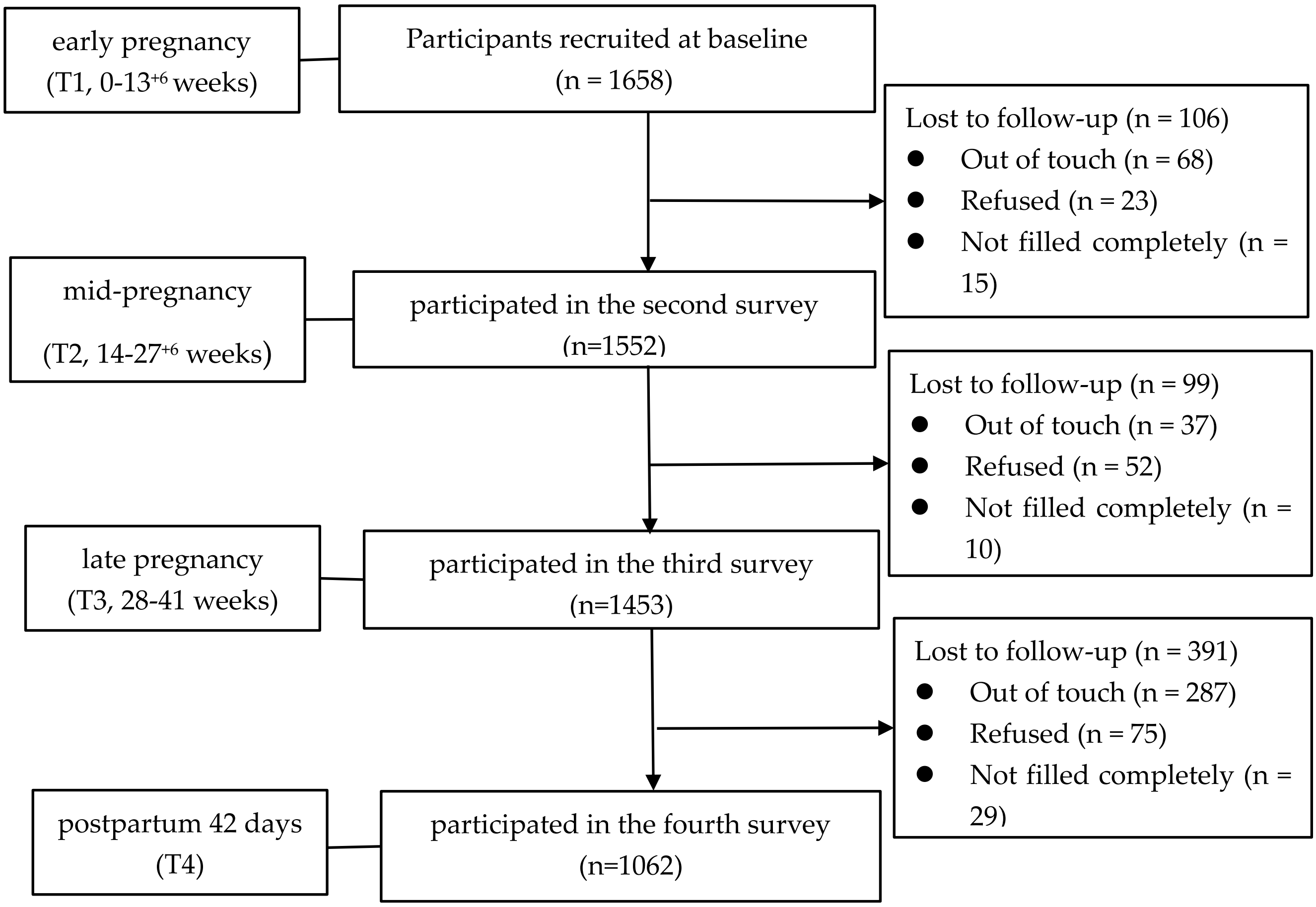

Figure S1. The flow chart of participant selection and follow-up

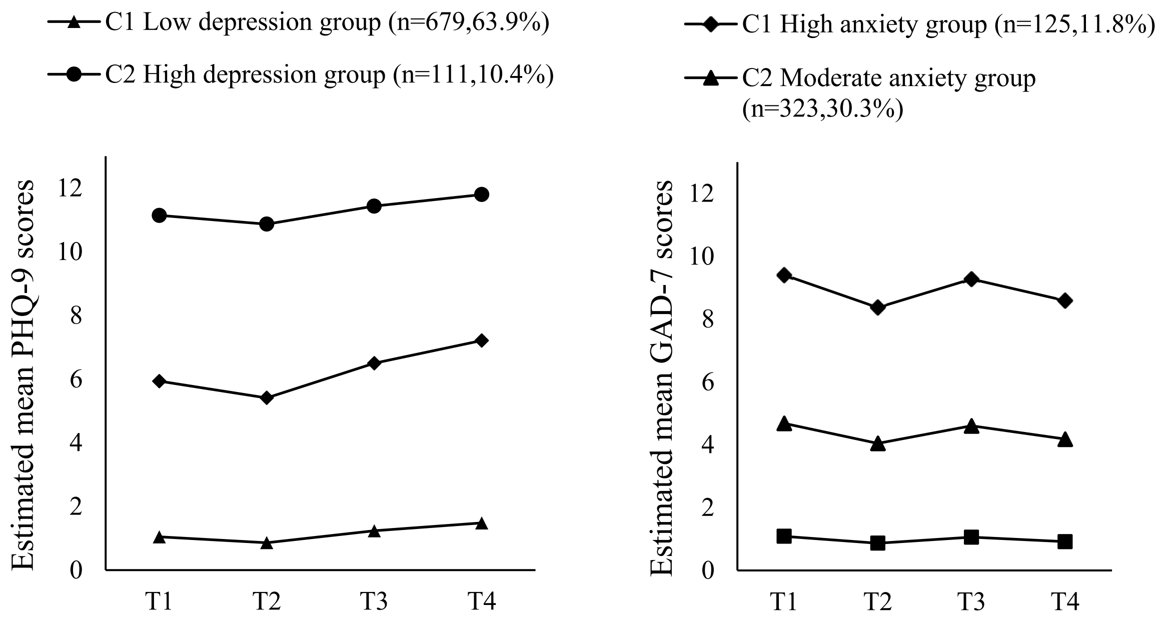

(**a**) (**b**)

Figure S2. (a) Developmental trajectory of perinatal depression in the three-class model; (b) Developmental trajectory of perinatal anxiety in the three-class model.

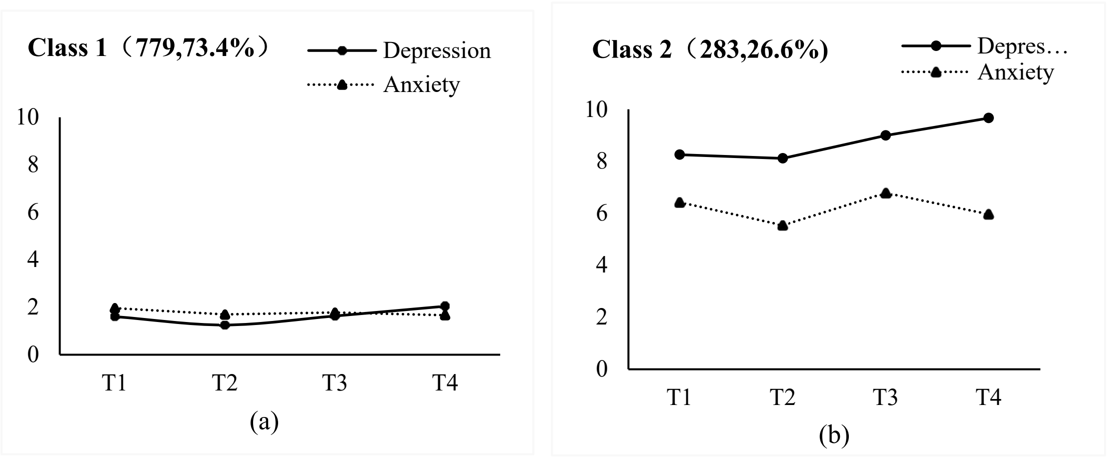

Figure S3. Joint developmental trajectories of perinatal depression and anxiety in the two-class model: (a) Class 1 "low-stable depression and anxiety group"; (b) Class 2 "high-increasing depression and high-slightly-decreasing anxiety group".
